# Molecular characterization of a 31 kDa protein from *Trichinella spiralis* and its induced immune protection in BALB/c mice

**DOI:** 10.1186/s13071-018-3198-5

**Published:** 2018-12-05

**Authors:** Hua Nan Ren, Kai Xia Guo, Yao Zhang, Ge Ge Sun, Ruo Dan Liu, Peng Jiang, Xi Zhang, Li Wang, Jing Cui, Zhong Quan Wang

**Affiliations:** 1 Department of Parasitology, Medical College, Zhengzhou University, 40 Daxue Road, Zhengzhou, 450052 People’s Republic of China; 2 Genetic and Prenatal Diagnostic Center of the First Affiliated Hospital, Medical College, Zhengzhou University, 40 Daxue Road, Zhengzhou, 450052 People’s Republic of China

**Keywords:** *Trichinella spiralis*, 31kDa protein, Larval invasion, Intestinal epithelium cells (IECs), ADCC, Immune protection

## Abstract

**Background:**

*Trichinella spiralis* is an important foodborne zoonotic parasite and it is necessary to develop a vaccine in order to interrupt transmission from animals to humans. A 31 kDa protein from *T. spiralis* (Ts31) is an antigen targeted by protective antibodies, and Ts31 contains a domain of trypsin-like serine protease that might have the function of serine protease. The purpose of this study was to investigate the molecular characteristics of Ts31 and its induced immune protection.

**Methods:**

Expression and localization of Ts31 in various *T. spiralis* phases were investigated using qPCR and immunofluorescent test (IFT). The specific binding between Ts31 and intestinal epithelium cells (IECs) was analyzed by Far-Western blotting, ELISA and IFT, and the cellular localization of binding sites was examined on confocal microscopy. The mice were subcutaneously vaccinated with recombinant Ts31 protein (rTs31), serum specific IgG was determined by ELISA, and immune protection induced by immunization with rTs31 was evaluated. Inhibition of anti-rTs31 IgG on IL1 invasion of IECs and ADCC-mediated killing of newborn larvae (NBL) was also determined.

**Results:**

Ts31 was expressed at different life-cycle stages and located principally at the stichosome and cuticle of this parasite. rTs31 was capable to specially bond to IECs, and binding site was located in the cytoplasm of IECs. Immunization of mice with rTs31 elicited a significant humoral response and protection, as demonstrated by a 56.93% reduction of adult worms at 6 days post-infection (dpi) and a 53.50% reduction of muscle larvae at 42 dpi after larval challenge. Anti-rTs31 antibodies impeded *T. spiralis* penetration of enterocytes in a dose-dependent pattern, and participated in the destruction of NBL by an ADCC-mediated manner.

**Conclusions:**

Ts31 facilitated the *T. spiralis* penetration of intestinal epithelium, which could make it a vaccine candidate target molecule against *Trichinella* infection.

## Background

Trichinellosis is a worldwide food-borne zoonosis caused by the parasitic nematodes *Trichinella* spp. [1]. Humans acquire this infection mainly by ingesting raw or poorly processed animal meat contaminated by muscle larvae (ML) of *Trichinella* [2, 3]. *Trichinella spiralis* is the principal causative agent of trichinellosis, and domestic pork is the primary infectious source of *Trichinella* infection in humans [4]. Cases with trichinellosis have been reported in 55 countries over the world, and many outbreaks of this disease were recorded, especially in developing countries [5, 6]. China is one of serious endemic areas of trichinellosis, and 15 trichinellosis outbreaks with four deaths occurred from 2004 to 2009 [7, 8]. Trichinellosis has an important impact on public health, pork food safety and socioeconomics [9].

*Trichinella spiralis* is an intracellular parasite with a direct life-cycle. Adult worms (AW) dwell in the small intestine of flesh-eating animals and ML in muscles of the same hosts. After infected meat is ingested, ML are liberated in the stomach and develop into intestinal L1 larvae (IL1) in the intestine at 0.9 h post-infection (hpi). The IL1 penetrate into the enteral epithelium where they molt four times and grow into AW [10, 11]. IL1 invasion of intestinal epithelium cells (IECs) is crucial for the establishment of *Trichinella* infection, whereas the intestinal epithelium is the first host’s native innate barrier combating *Trichinella* infection and the principal location for the interaction between the intestinal nematode and the host [12, 13]. If the IL1 invasion of IECs, larval development and newborn larvae (NBL) deposition are interrupted, or IL1, AW and NBL are expelled from the intestine by vaccination, *Trichinella* infection will be prevented or eliminated in the early stage of enteral infection [14, 15]. Therefore, development of an anti-*Trichinella* vaccine is necessary for the control and elimination of porcine *Trichinella* infection to ensure pork safety [16–18].

*Trichinella spiralis* excretory/secretory (ES) antigens are exposed to the host immune system and play a significant role in regulating host immune responses and parasitism [19]. By proteomics analysis some serine proteases have been identified in *T. spiralis* larva ES proteins [20, 21]. Serine proteases secreted by parasite might have a crucial role in the modulation of host-parasite interactions, for example mediating or facilitating invasion, taking nutrition, or evading the host’s immune responses [22–24]. Serine proteases could be potential vaccine target molecules against *T. spiralis* infection.

In our previous studies, a 31 kDa protein from *T. spiralis* (Ts31, i.e. antigen targeted by protective antibodies, GenBank: AAA20539) was identified from ML ES proteins by immunoproteomics [25, 26]. It was also found in IL1 and AW ES proteins probed by infection sera [27, 28]. Bioinformatics analysis indicated that the complete Ts31 sequence was 858 bp with one open reading frame (ORF) encoding 285 amino acids, with 31.3 kDa and pI 6.1. We cloned and expressed Ts31 and the recombinant Ts31 protein (rTs31) had the potential for diagnosis of trichinellosis [29].

Since Ts31 has a domain of trypsin-like serine protease with an active site carrying a classic catalytic triad for proteolysis, it might have the function of serine protease. The purpose of this study was to analyze the characteristics and functions of the Ts31 and to investigate the immune protection elicited by vaccination with rTs31.

## Methods

### Parasites and experimental animals

The *T. spiralis* strain (ISS534) used in our study was isolated from domestic pigs in Henan Province of China. We maintained this strain by serial passage in BALB/c mice. Female BALB/c mice were obtained from Henan Provincial Experimental Animal Center (Zhengzhou, China) and given free access to food and water.

### Different stage worms

ML were collected by artificial digestion of *T. spiralis*-infected mouse carcasses at 42 dpi [30, 31]. IL1 were isolated from mouse intestines at 6 hpi [32] and AW were recovered from the intestines at 3, 5 and 6 dpi [33]. The AW at 5 dpi were washed with sterile PBS and cultured at 37 °C for 24 h in RPMI-1640 (50 worms/ml) and the NBL were collected [34]. The IL1 ES proteins were prepared as described [35].

### Cell culture and protein preparation

The primary IECs were separated from mouse intestines and susceptible to the IL1 invasion [11]. Mouse striated muscle myoblast C2C12 cells were unsusceptible to the IL1 invasion and served as negative control [36, 37]. These cells were cultivated in Dulbecco’s modified Eagle’s media (DMEM; Gibco, Thermo Fisher Scientific, Waltham, MA, USA) containing 5% fetal bovine serum (FBS; Gibco) and collected by trypsinization [38]. The lysates of IECs and C2C12 cells were prepared as described previously [17].

### Preparation of the rTs31 and mouse anti-rTs31 serum

The Ts31 gene was cloned and recombinant expression plasmid pMAL-c2X/Ts31 was transformed into *Escherichia coli* TB1. The recombinant Ts31 protein (rTs31) was induced for 6 h at 30 °C with 0.3 mM IPTG, expressed as soluble protein with a N-terminal maltose-binding protein (MBP) tag, and purified using affinity chromatography with amylose resin (New England Biolabs, Ipswich, MA, USA) in our laboratory [29]. The rTs31 protein concentration was then assayed [39].

Fifteen mice were subcutaneously immunized with 20 μg of rTs31 emulsified with complete Freund’s adjuvant, then boosted three times using rTs31 with incomplete Freund’s adjuvant at a two-week interval [40]. Anti-rTs31 serum was collected from immunized mice at two weeks following the final immunization; pre-immune serum was used as negative control.

### Real-time quantitative PCR (qPCR)

Total RNA from various stage worms (ML, IL1, 3 and 6 dpi AW, and NBL) was isolated with Trizol reagent (Invitrogen, Carlsbad, CA, USA). The transcriptional level of Ts31 at various stage worms was determined by qPCR [12, 38]. The qPCR specific primers for Ts31 gene were 5'-TGT CAG TGT CGG TTC TCC TG-3' and 5'-CAT CTG GTA AAG GAA CGC TTG C-3'. The transcription level of Ts31 was normalized by subtracting the transcription level of a *T. spiralis* housekeeping gene GAPDH (GenBank: AF452239) and the then calculated on the basis of the comparative Ct (2−ΔΔCt) method [41]. Each experiment was performed three times and each sample had three replicates.

### Immunofluorescent test (IFT)

The tissue location of Ts31 at *T. spiralis* worms at various stages (ML, IL1 and 3d AW) was observed by IFT [23, 42]. Worm sections with 3 μm thickness were prepared by a microtome, blocked at 37 °C for 1 h with 5% normal goat serum. Mouse anti-rTs31 serum (1:10 dilution) served as the primary antibodies. After washing, the sections were stained with 1:200 dilutions of anti-mouse IgG-FITC conjugate (Santa Cruz Biotechnology, Dallas, TX, USA). After washing again, the sections were observed under fluorescent microscopy (Olympus, Tokyo, Japan) [43].

### Far-Western blotting

To investigate the interaction between rTs31 and IECs *in vitro*, soluble proteins of IEC and C2C12 were analyzed on SDS-PAGE with 12% gels [21]. The proteins were then transferred to nitrocellulose membranes (Merck Millipore, Billerica, MA, USA) then the membrane was sliced and blocked with 5% skim milk in PBS-0.5% Tween 20 (PBST) for 1 h at 37 °C. Subsequently, the blots were incubated with 20 μg/ml rTs31 or MBP and PBS control for 1 h at 37 °C [38, 44]. After washing, the blots were probed with 1:100 dilutions of anti-rTs31 serum, infection serum or pre-immune serum at 37 °C for 1 h. After washing thoroughly, the membranes were incubated (37 °C, 1 h) with anti-mouse IgG-HRP conjugate, and colored with 3,3′-diaminobenzidine (DAB) (Sigma-Aldrich, St. Louis, MO, USA). The protein bands of IECs binding with rTs31 were analyzed by AlphaView software (AIC) [45].

### Assay of the binding of Ts31 with IECs by ELISA

The capability of rTs31 binding to IEC was measured by ELISA [17]. The plate was coated with various concentration of IEC proteins (0.16, 0.32, 0.64, 1.28, 2.56, 5.12 and 10.24 μg/ml) overnight at 4 °C. After blocking and washing, the plate was incubated with different concentration of rTs31 (1, 2, 3, 5, 10 and 15 μg/ml), and then with different mouse sera (1:100; 37 °C, 2 h). HRP labelled anti-mouse IgG conjugate (1:10000; Sigma-Aldrich) was used as secondary antibodies, and then colored by using o-phenylenediamine dihydrochloride (OPD; Sigma-Aldrich) as the substrate. Absorbance at 490 nm was measured for each sample [35].

### Cell immunostaining and confocal microscopy

IEC and C2C12 were cultured on glass coverslips in DMEM medium [46]. When the cells were grown to confluence, the cell monolayer was co-cultured at 37 °C for 2 h with 20 μg/ml rTs31; IL1 ES protein, MBP and PBS were used as controls. The monolayer was fixed in 4% phosphate-buffered paraformaldehyde and blocked at 37 °C for 1 h with 5% goat serum. The monolayer was probed using a 1:10 dilution of anti-rTs31 serum, pre-immune serum or infection serum at 37 °C for 2 h. After washing, the monolayer was dyed using anti-mouse IgG-FITC conjugate (1:100, Santa Cruz Biotechnology) and cell nuclei were dyed by propidium iodide (PI) for 5 min [47]. Finally, the cellular localization of rTs31 within IECs was examined under time-lapse confocal microscopy [38].

### Assay of the *in vitro* larval invasion of IECs

To investigate the inhibition of anti-rTs31 serum on the larval invasion of IEC, the ML were activated into IL1 using 5% mouse bile (1:20) and utilized for the invasion test [12, 48]. The IEC monolayer was covered with 200 IL1 suspended in 2 ml of DMEM semisolid media with 1.75% agarose. The media were pre-added with anti-rTs31 serum (1:50 to 1:1000), 1:50 dilutions of pre-immune serum or infection serum [49]. After being incubated for 2 h at 37 °C, the IL1 penetration of the IEC monolayer was observed and numbered under microscopy. The larvae invading and migrating in monolayer were regarded as invaded larvae, whereas the larvae suspended in media were taken as non-invaded larvae [42]. Three independent tests for three kinds of serum were performed and three repeats served to evaluate the larva invasion rate for each kind of serum [38].

### Antibody-dependent cellular cytotoxicity (ADCC) test

A test of the cytotoxic effect of anti-rTs31 antibodies on the NBL was performed [50, 51]. Briefly, 150 NBL of *T. spiralis* were incubated at 37 °C for 60 h with 2 × 10^5^ peritoneal exudates cells (PECs) from uninfected mice in the presence of anti-rTs31 serum diluted at 1:5–1:1000 with RPMI 1640 media. Each test was performed in triplicate. The morphology and viability of larvae treated by ADCC were observed *via* microscopy. Live NBL were defined as active and exhibiting wriggling movement, whereas dead NBL were defined as rigid, immobile or disintegrated [41, 52]. Moreover, the dead larvae were usually adhered by clumps of PECs. The results are shown as the percentage of dead NBL to the total NBL examined in each assay.

### Immunization regimen and detection of anti-rTs31 antibodies

Eighty mice were divided into four groups (20 animals per group). Mice of the immunized group were subcutaneously vaccinated with 20 μg of rTs31 as the same immunization regimen as mentioned in the preparation of mouse anti-rTs31 serum [40]. Mice of three control groups were subcutaneously injected with only MBP-tag protein, adjuvant alone, or PBS, based on the same vaccination scheme. About 50 μl of tail blood was obtained from all vaccinated mice two weeks after each vaccination. Mouse pre-immune serum was also collected and used as a negative control [53].

Serum anti-rTs31 antibodies (total IgG, IgG1 and IgG2a) in immunized mice were measured using ELISA [54, 55]. Microtiter plate (Nunc, Roskilde, Denmark) was coated using rTs31 (2 μg/ml) overnight at 4 °C, and blocked at 37 °C for 1 h using 5% skim milk. Immune sera were diluted 1:100 and incubated at 37 °C for 2 h. After washing, the plate was incubated with HRP labelled anti-mouse IgG, IgG1 or IgG2a conjugate (1:5000; Sigma-Aldrich) for 1 h at 37 °C. The coloration by using OPD and determination of the absorbance at 490 nm were performed as previously described [35].

### Larval challenge experiment

To assess the immune protection of rTs31, each mouse of four groups of vaccinated mice (20 animals per group) was orally inoculated with 300 *T. spiralis* ML at 2 weeks after last vaccination. Ten mice from each group were killed at 6 dpi and AW were examined. The ML burden of the other 10 mice from each group was examined at 42 dpi [54]. The immune protection was estimated based on the number of AW or larvae per gram (LPG) of skeletal muscles collected from the immunized group compared to those from the PBS group [16, 56].

### Statistical analysis

All statistical analyses were performed with SPSS for Windows, version 20.0 (SPSS Inc., Chicago, IL, USA). Data are shown as the means ± standard deviation (SD). Difference among the different groups was analyzed using a Chi-square test, Student’s t-test or one-way ANOVA. *P* < 0.05 was regarded as statistically significant.

## Results

### Transcription level of the Ts31 gene at different stage worms

**Fig. 1:**
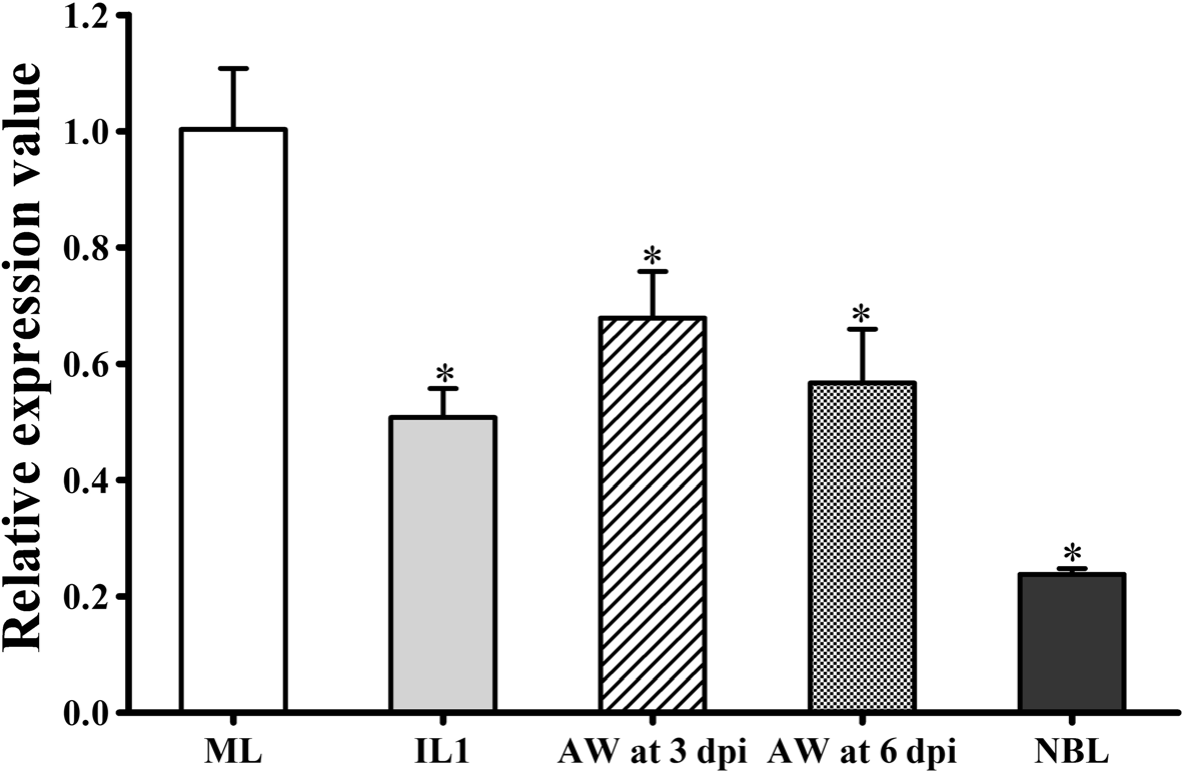
Ts31 transcription level at *T. spiralis* different stage worms by qPCR. The mRNAs from *T. spiralis* different stage (ML, IL1, 3 and 6 dpi AW and NB) were subjected to qPCR. Asterisks indicate significant differences compared with the ML stage (*P < 0.001)

qPCR analysis revealed that the Ts31 gene was transcribed at different life-cycle stages for *T. spiralis* worms (ML, IL1, 3 and 6 dpi AW, and NBL) (Fig. 1). The transcriptional level of Ts31 at the ML stage was significantly higher than those of other worm stages (*F*_(4,10)_ = 40.776, *P* < 0.001).

### Expression and localization of Ts31 at different stage worms

**Fig. 2:**
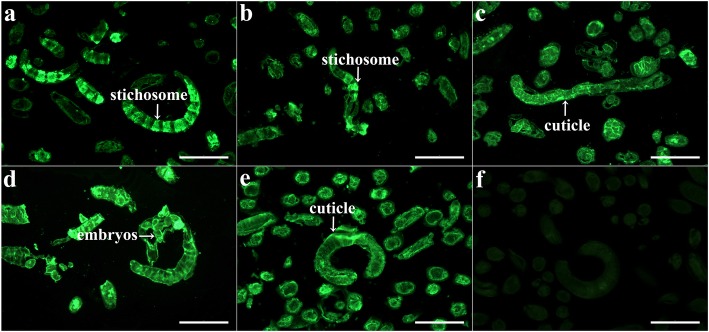
Tissue location of Ts31 at various *T. spiralis* stages by IFT using anti-rTs31 serum*.* Immunostaining is distributed at stichosome of ML (**a**) and IL1 (**b**), cuticle of 3 dpi male (**c**), and embryos of 3 dpi female (**d**). The ML incubated with infection serum was used as a positive control (**e**), and ML incubated with pre-immune serum as negative controls (**f**). *Scale-bars*: 100 μm

The IFT results indicated that the immunofluorescent staining was observed at different stages (ML, IL1, female and male at 3 dpi) by using anti-rTs31 serum. Immunostaining was located at the cuticle, stichosome of ML, IL1, 3 dpi male and embryos of 3 dpi female (Fig. 2). No fluorescence staining was seen in ML incubated with pre-immune serum.

### Far-Western blotting analysis of rTs31 binding to IECs

**Fig. 3:**
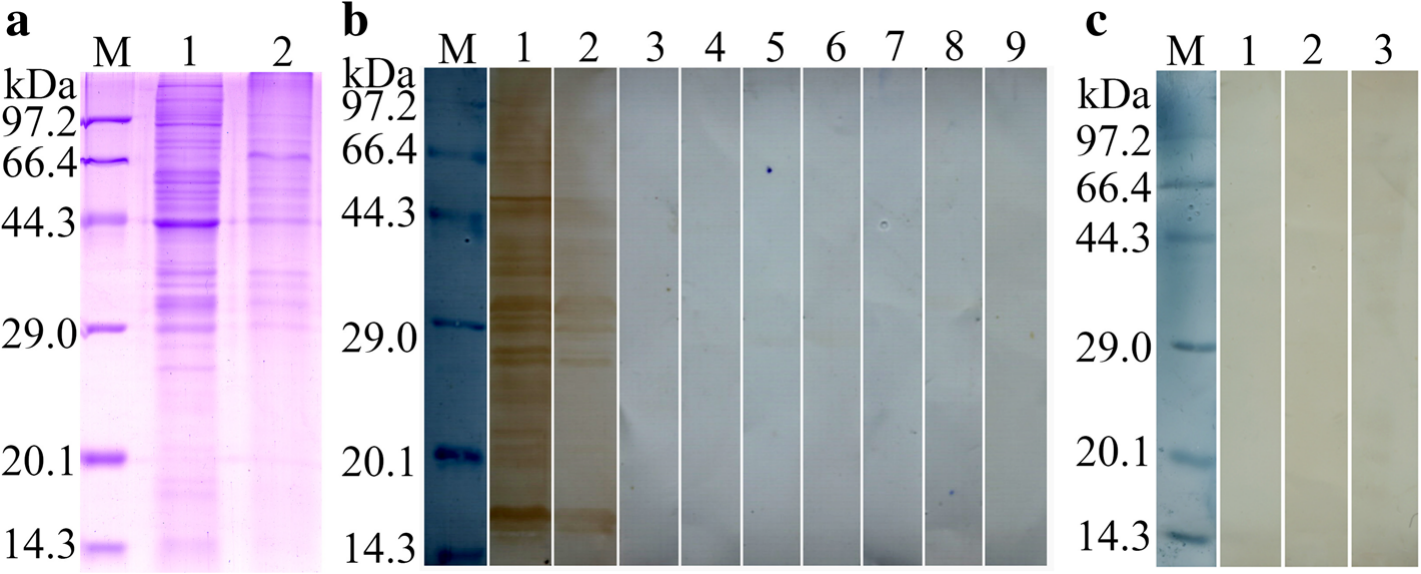
Binding between rTs31 and IECs analyzed by Far-Western blotting. **a** SDS-PAGE of IEC (Lane 1) and C2C12 (Lane 2) lysates; Lane M: protein marker. **b** Far-Western blotting of IEC proteins bound with rTs31. The IEC proteins were firstly pre-incubated with rTs31 (Lanes 1–3), MBP (Lanes 4–6) or PBS (Lanes 7–9), and subsequently probed with anti-rTs31 serum (Lanes 1, 4 and 7), infection serum (Lanes 2, 5 and 8) or pre-immune serum (Lanes 3, 6 and 9). **c** Far-Western blotting of C2C12 proteins bound with rTs31. The C2C12 protein (Lanes 1–3) was first pre-incubated with rTs31 and not recognized by anti-rTs31 serum (Lane 1), infection serum (Lane 2) or pre-immune serum (Lane 3). There was no binding between rTs31 and C2C12 protein

SDS-PAGE results revealed that the IEC lysates have about 31 protein bands of 14.3–97.2 kDa (Fig. 3a). Regarding Far-Western blotting, approximately 27 bands (15.3–89.8 kDa) of IEC proteins pre-incubated with rTs31 were recognized by anti-rTs31 serum; about 11 bands (15.8–47.9 kDa) were identified by using infection serum. The IEC proteins pre-incubated with rTs31 were not recognized by pre-immune serum. The IEC proteins pre-incubated with MBP or PBS were not recognized by anti-Ts31 serum or infection serum (Fig. 3b). There was no detectable binding between the C2C12 proteins and rTs31 (Fig. 3c). These results demonstrated that there was a specific binding and interaction between rTs31 and IECs.

### Protein binding of rTs31 with IECs measured by ELISA

**Fig. 4:**
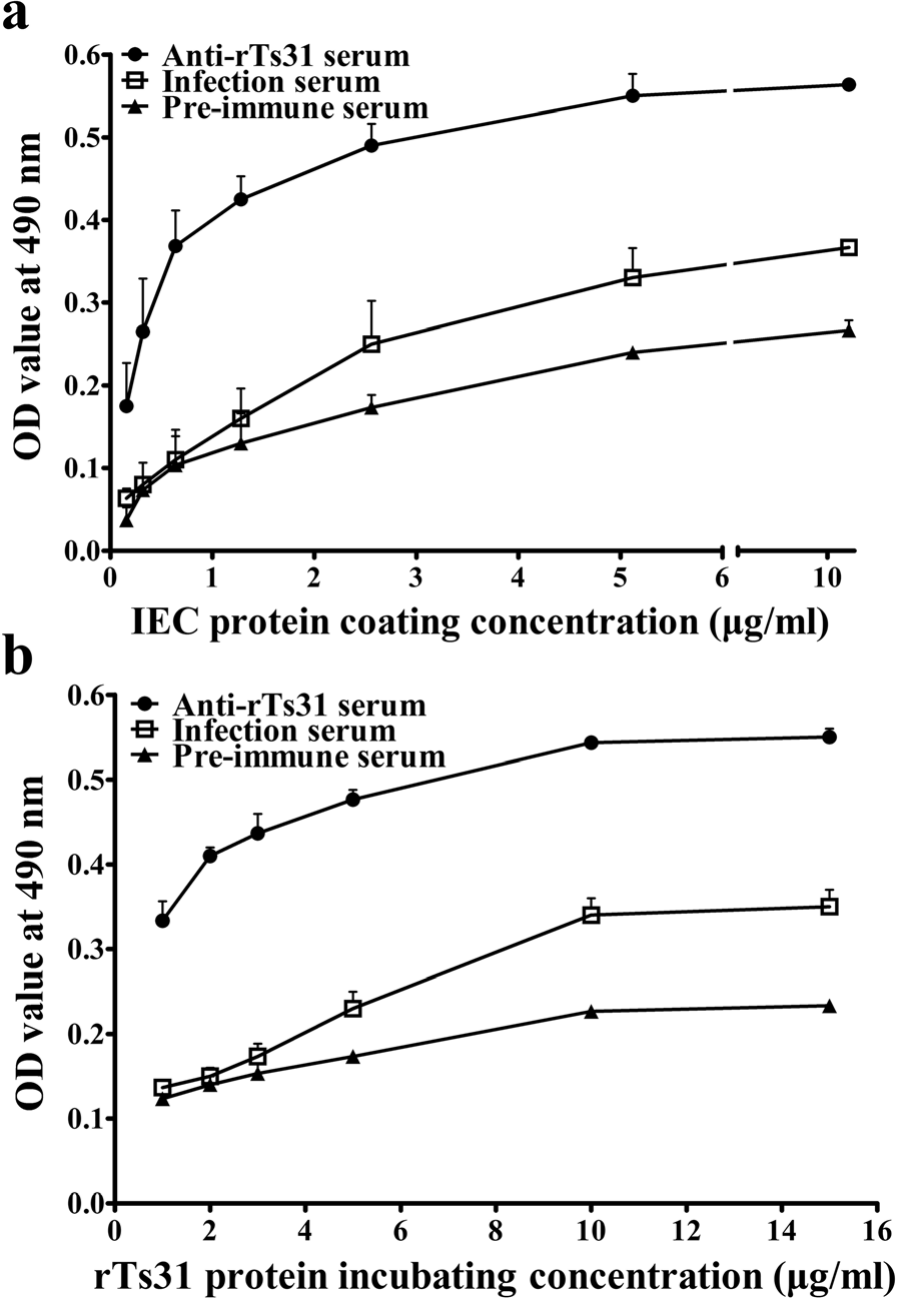
Protein binding activity of rTs31 with IECs measured by ELISA. **a** Binding between IEC protein at different concentration and rTs31 (10 μg/ml). **b** Binding between IEC protein (6 μg/ml) and rTs31 at different concentration. The binding of rTs31 with IECs is dose-dependent on both rTs31 and IEC proteins

ELISA results revealed that there was an evident protein binding activity between rTs31 and IEC proteins. The OD value was dose-dependent on IEC proteins (*r*_(6)_ = 0.757, *P* < 0.001) and revealed an elevating trend with increasing IEC protein concentration (*F*_(6,14)_ = 41.086, *P* < 0.001) (Fig. 4a). Furthermore, the OD value was also rTs31 dose-dependent (*r*_(5)_ = 0.888, *P* < 0.001) and displayed an elevating trend with increasing rTs31 concentration (*F*_(5,12)_ = 86.386, *P <* 0.001) (Fig. 4b).

### Binding of rTs31 with IECs and cellular localization

**Fig. 5:**
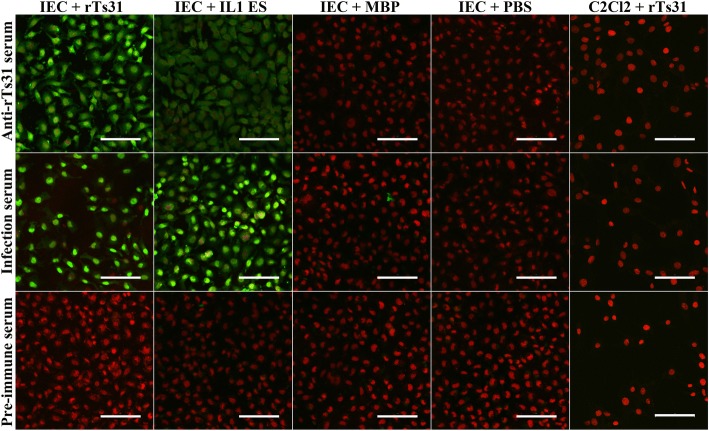
IIF analysis of rTs31 binding to IECs. The IECs were first co-cultured with rTs31, IIL ES antigens, MBP or PBS. C2C12 cells were also co-cultured with rTs31. After blocking and washing, the IECs and C2C12 was probed by anti-rTs31 serum, infection serum or pre-immune serum, and subsequently stained using anti-mouse IgG-FITC conjugate. Cell nuclei were dyed by propidium iodide (PI) as the red. *Scale-bars*: 100 μm

**Fig. 6:**
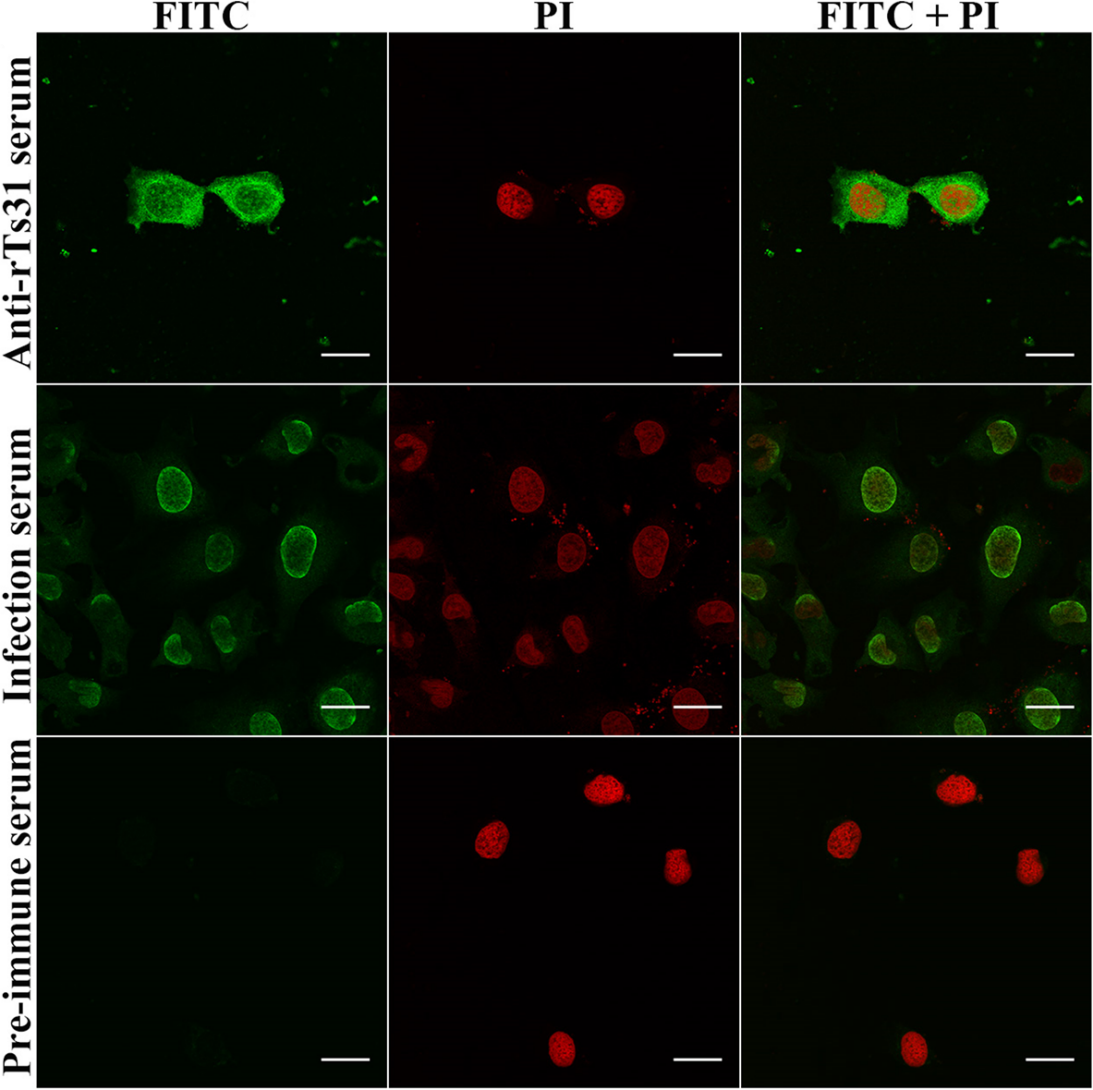
Cellular localization of rTs31 in IEC cells under confocal microscopy. The IEC cells were first co-incubated with rTs31, then reacted with anti-rTs31 serum, infection serum or pre-immune serum, and finally dyed with FITC-anti-mouse IgG conjugate. Propidium iodide (PI) stained cell nuclei in red. *Scale-bars*: 5 μm

IFT results demonstrated that after IECs were pre-incubated with rTs31 or IL1 ES antigens, fluorescent staining was detected on IECs probed by anti-rTs31 serum or infection serum, but not by pre-immune serum. The IECs pre-incubated with MBP or PBS did not reveal any staining due to anti-rTs31 serum or infection serum. No staining on C2C12 cells was seen by using anti-rTs31 serum or infection serum (Fig. 5). Confocal microscopy revealed the staining was distributed in the cytoplasm of IECs, especially around nucleus (Fig. 6), demonstrating that rTs31 was capable of specifically binding to the IEC and entering the cytoplasm.

### Inhibition of *T. spiralis* invasion of IECs by anti-rTs31 serum

**Fig. 7:**
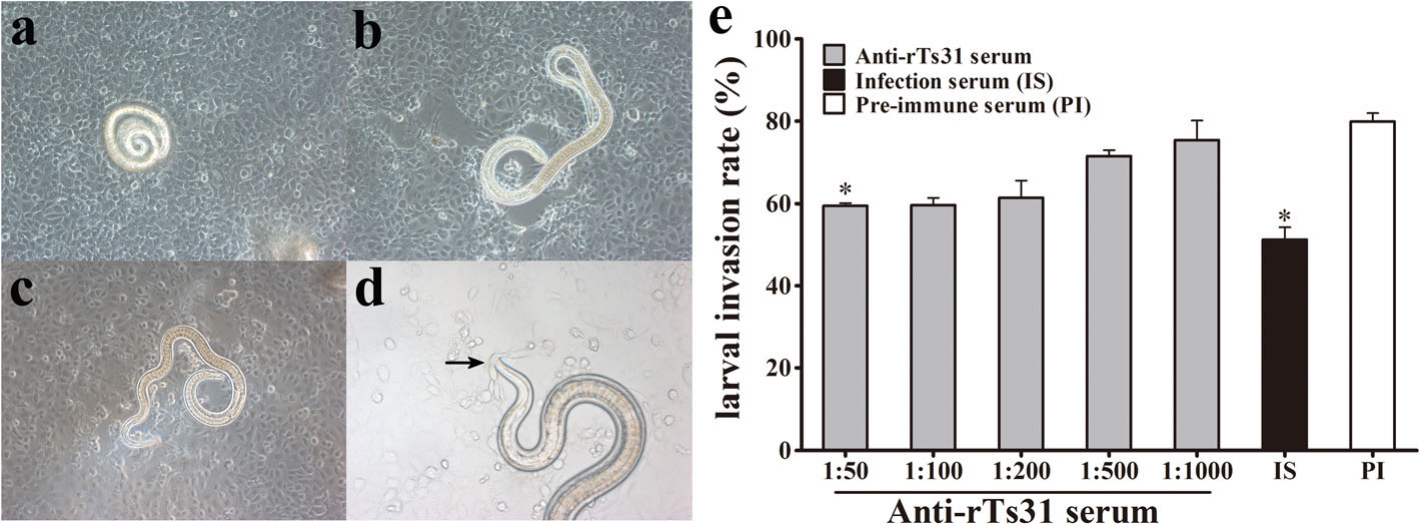
The *in vitro* inhibition of *T. spiralis* invasion of IEC monolayer by anti-rTs31 serums. When the IEC monolayer was overlaid by semisolid media containing 200 IL1 larvae and cultured at 37 °C for 2 h, non-invaded (**a**) and invaded larvae (**b** and **c**) in the monolayer were observed (200×). The larva head is penetrating into the IEC monolayer as indicated by the arrow (**d**, 400×). **e** Inhibition of different dilutions of anti-rTs31 serum on larval invasion. Infection serum (IS, 1:50 dilution) and pre-immune serum (PI, 1:50) were used as positive and negative controls, respectively. The data are presented as percentages of the larvae invaded in IECs out of all larvae used in each assay and expressed as the mean ± SD of three independent assays. Asterisks indicate significant differences (*P < 0.001) in comparison with pre-immune serum group

The IEC monolayer was overlaid by semisolid media containing 200 IL1 larvae and cultured for 2 h. During this time, the larvae invaded and migrated in the IEC monolayer (Fig. 7a-d). When the media were replenished by 1:50 dilutions of anti-rTs31 serum, pre-immune serum or infection serum, and incubated for 2 h, the percentage of larvae penetrating into the monolayer was 59.48, 80.04 and 51.25%, respectively (Fig. 7e). The inhibition of anti-rTs31 serum on larval invasion of the monolayer was more significant than that of pre-immune serum (*χ*^2^_(1)_ = 18.432, *P* < 0.001), and the inhibition was dose-dependent of anti-rTs31 antibodies and exhibited a reducing trend with serum dilution elevation (*F*_(4,10)_ = 18.797, *P* < 0.001). However, we did not observe a significant inhibition of larva invasion of IECs by using pre-immune serum.

### Killing of NBL mediated by ADCC

**Fig. 8:**
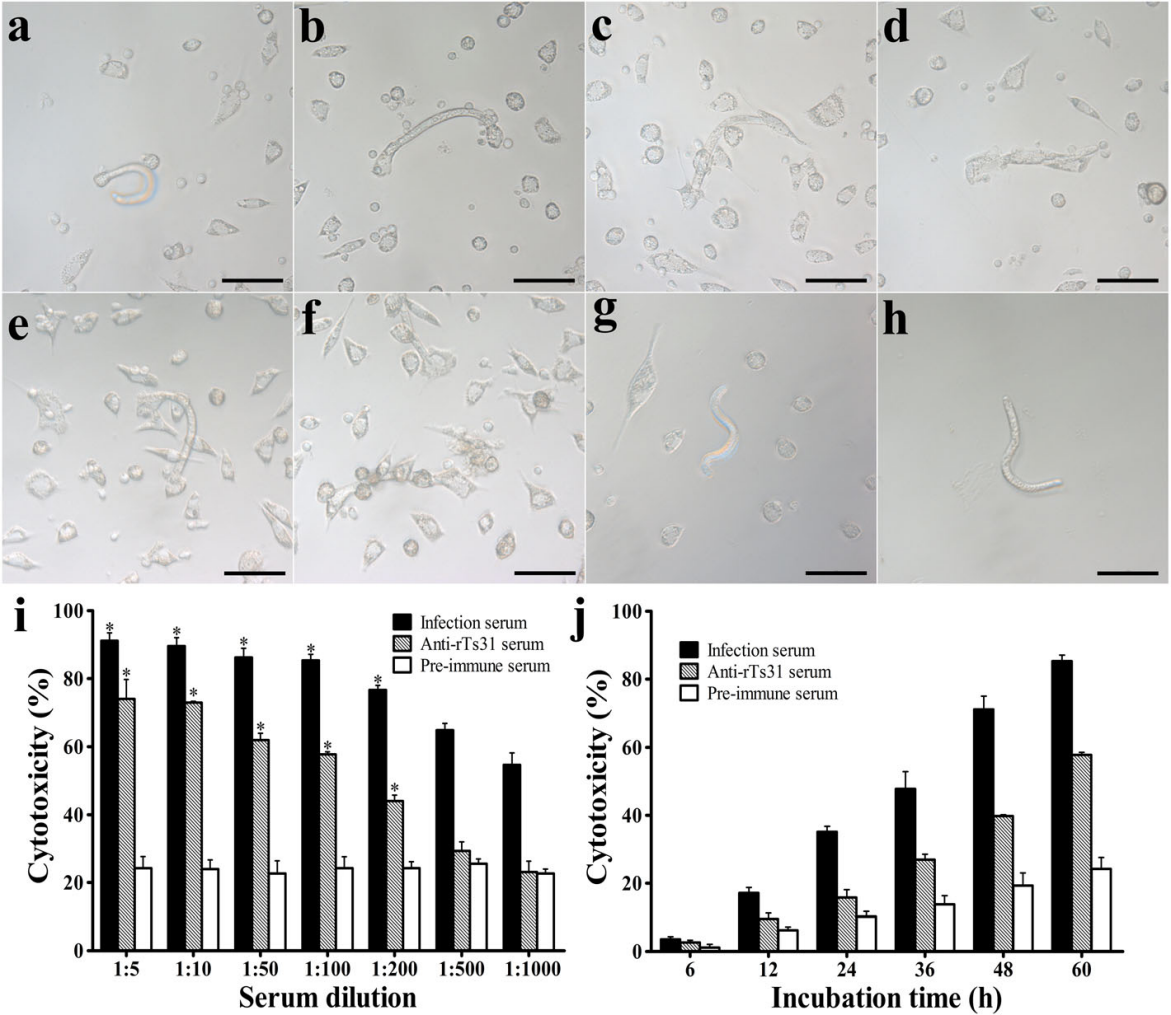
Killing of NBL mediated by ADCC. **a**-**f** Morphology of *T. spiralis* NBL treated by ADCC test. The NBL (**a-d**) were treated using anti-rTs31 serum and 2 × 10^5^ mouse PECs at 37 °C for different times: **a** 24 h, **b** 36 h, **c** 48 h and **d** 60 h. **e-f** NBL treated by infection serum for 48 h (**e**) and 60 h (**f**). **g** NBL treated using pre-immune serum for 60 h. **h** NBL incubated without PECs for 60 h. **i** The cytotoxicity was dose-dependent on anti-rTs31 antibodies. **j** The cytotoxicity had an evelating trend with culture time prolongation. Asterisks indicate significant differences (*P < 0.001) in comparison to cytotoxicity of pre-immune serum. *Scale-bars*: 50 μm

After incubation for different times, anti-rTs31 antibodies mediated and facilitated the adhering and killing of PECs to NBL (Fig. 8). When anti-rTs31 serum (1:100) was incubated with the NBL and PECs for 60 h, the ADCC induced statistically significant NBL death (57.78%, cytotoxicity) relative to NBL treated with pre-immune serum (24.22%, cytotoxicity) (*χ*^2^_(1)_ = 35.841, *P* < 0.001). When different dilutions of anti-rTs31 serum were used to incubate with the NBL and PECs for 60 h, the cytotoxicity was dose-dependent on anti-rTs31 antibodies (*r*_(6)_ =0.973, *P* < 0.001) and showed a reducing trend with the elevation of serum dilution (*F*_(6,14)_=150.53, *P* < 0.001). A significant correlation between cytotoxicity and culture time was also observed (*r*_(5)_=0.978, *P* < 0.001) and the cytotoxicity showed an elevating trend with increasing the culture time (*F*_(5,12)_=620, *P* < 0.001) (Fig. 8).

### Anti-rTs31 antibody response triggered by vaccination with rTs31

**Fig. 9:**
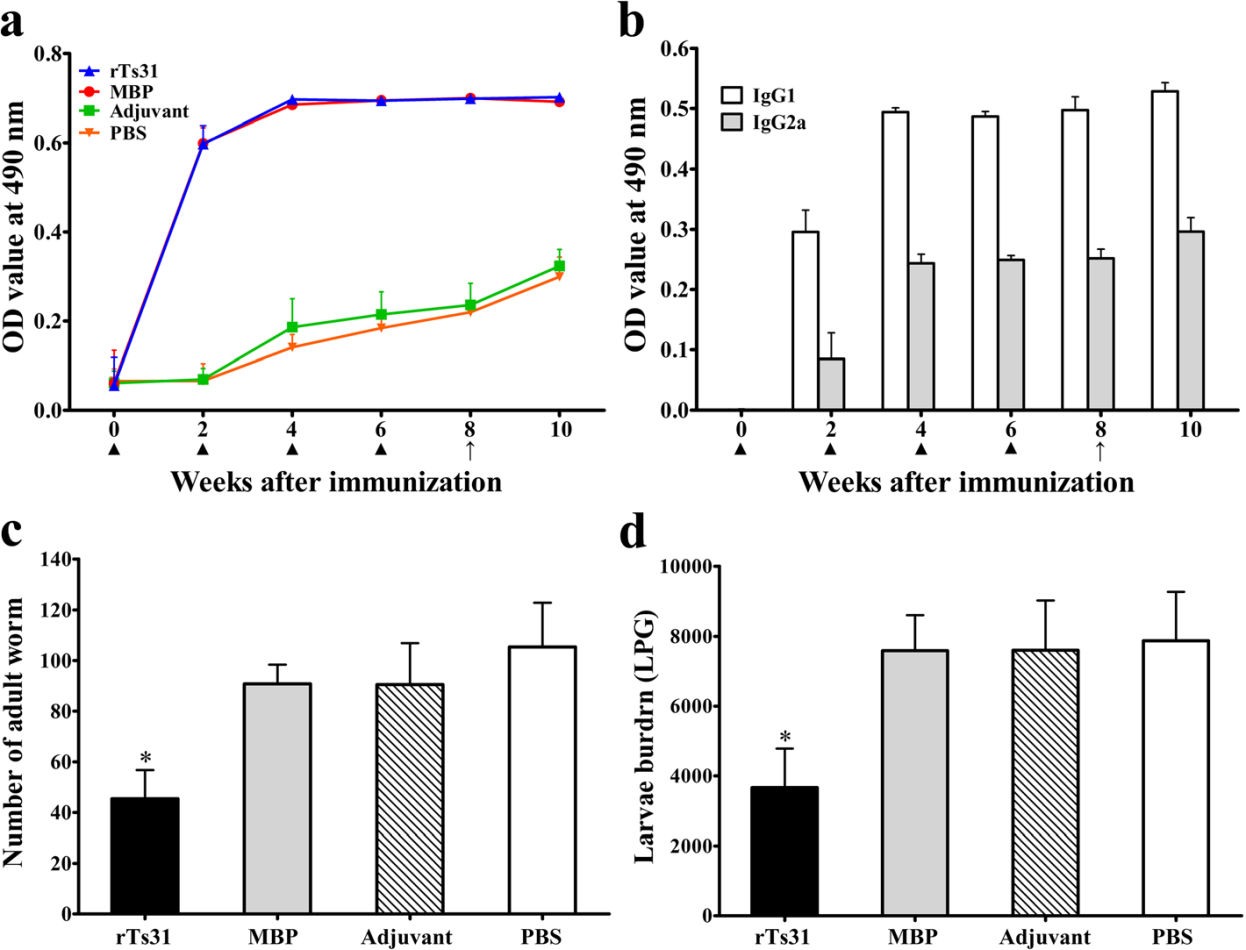
Anti-rTs31 antibody responses and immune protection in vaccinated mice. **a** Total anti-rTs31 IgG in serum of mice vaccinated with rTs31 (MBP tag, adjuvant or PBS) at various times post-vaccination. **b** Specific IgG subtypes (IgG1 and IgG2a) responses to rTs31 at various times post-vaccination. The OD value from each group is the mean ± SD of the antibody levels (*n* = 10). The vaccination time is indicated by solid triangle, and the challenge time is indicated by a solid arrow. **c** and **d** Adult worm (**c**) and muscle larvae (**d**) burden of vaccinated mice after larval challenge. The data are presented as the mean ± SD of each group (*n* = 10). Asterisks indicate a statistical difference (*P < 0.001) in worm burden in rTs31-immunized group relative to the control groups (MBP, adjuvant and PBS group)

In order to assess anti-rTs31 antibody response, anti-rTs31 IgG, IgG1 and IgG2a were measured by ELISA at various times following vaccination. The serum anti-rTs31 IgG level of mice vaccinated with rTs31 was dramatically raised after the first vaccination (Fig. 9a). Nevertheless, the mice inoculated with MBP also exhibited the same high level of total IgG, which was likely due to the rTs31 containing MBP tag proteins. However, all mice injected with adjuvant alone or PBS did not evidently display anti-rTs31 antibody responses. The IgG1 levels on weeks 2, 4, 6 and 8 post-vaccination were remarkably higher than for IgG2a (*t*_2w(18)_ = 11.845, *t*_4w(18)_ = 48.085, *t*_6w(18)_ = 66.659, *t*_8w(18)_ = 30.570, *P* < 0.001). IgG2a was also triggered following the second immunization (Fig. 9b). Nevertheless, anti-rTs31 IgG and its subclass levels did not continue to elevate following larval challenge infection. The results demonstrated that a high level of specific anti-rTs31 antibodies was elicited in mice vaccinated with rTs31.

### Immune protection of rTs31

Immunization of mice with rTs31 displayed a 56.93% AW reduction and a 53.50% ML reduction (Fig. 9c-d), compared to that of the PBS group (*t*_adults (18)_ = 9.116, *t*_larvae (18)_ = 7.463, *P* < 0.001). Furthermore, AW and ML burdens from immunized mice were also significantly lower than those of the adjuvant group (*t*_adults (18)_ = 7.162, *t*_larvae (18)_ = 6.873, *P* < 0.001) or MBP tag group (*t*_adults (18)_ = 10.53, *t*_larvae (18)_ = 8.214, *P* < 0.001).

## Discussion

Serine proteases are one of the most important families of proteases and perform the principal functions in parasitic infection, such as larval invasion, molting, digestion and proteolysis, and regulation of development [24]. In parasitic helminths, it is known that serine proteases participate in parasite invasion of host tissues and play a major role in nematode molting [57]. Previous studies revealed that *Trichuris muris* serine proteases can hydrolyze the host’s mucus barrier, which prevents clearance of this nematode from the gut [58]. A serine protease from *Onchocerca volvulus* ES products can degrade the components of collagen type IV, dermal extracellular matrix, fibronectin and laminin, suggesting that the serine protease participates in the degradation of elastic fibres of the host tissue based on its proteolytic activity [59]. A serine protease from *Schistosoma mansoni* might participate in the cercarial invasion of host skin [60].

Serine proteinases from *Trichinella spiralis* AW have been identified from their ES products; the purified serine proteinases exhibited enzyme activity against gelatin and azocasein, and the enzymatic activity could be inhibited by the IgG purified from serum of infected hosts, which might be related with the damage of *Trichinella* survival in host [61]. On immunolocalisation analysis, a serine protease from *Trichinella spiralis* (TsSerP) was located on the inner layer of the cuticle and oesophagus of the parasite, suggesting it might have a potential role in molting or digesting [62]. Another *T. spiralis* serine protease (TspSP1.2) was cloned and expressed; anti-rTspSP1.2 serum inhibited *T. spiralis* invasion of IECs, and immunization of mice with rTspSP1.2 produced significant protective immunity [23].

In the present study, Ts31 was expressed in *E. coli*. qPCR results revealed that Ts31 mRNA was transcribed at all *T. spiralis* stages (ML, IL1, AW and NBL) and the Ts31 transcriptional level at the ML stage was significantly higher than that of the other stages, which might be related with worm age and their different living environments. For example, the AWs lodge in the host’s intestine and persist for 10–20 days in mice and rats or 4–6 weeks in humans whereas the ML parasitize in the host’s skeletal muscle for 1–2 years, or even up to 10–15 years without any major harm [10]. Ts31 was primarily distributed at the cuticle and stichosome (secretory organ) of the nematode, suggesting that Ts31 is an ES protein. The results are consistent with the fact that Ts31 was identified in ES proteins of *T. spiralis* ML, IL1 and AW [26–28], and the ES proteins principally derived from the shed cuticle proteins and stichosome secretory granules of this parasite [26, 32, 63]. This characteristic of Ts31 is similar to other *T. spiralis* serine proteases TsSerP and TspSP1.2 [23, 24]. *Trichinella spiralis* serine proteases might have housekeeping functions and might be an indispensable protease in larval invasion, development and survival in the host. Additionally, sequence analysis shows that the Ts31 contains a domain of trypsin-like serine protease [29]. rTs31 was found to have no serine protease activity, as determined through gelatin zymography (data not shown). The inactivity as a serine protease for the rTs31 in this study may result from the improper folding expressed in prokaryotic cells. Further expression of rTs31 in a eukaryotic expression system such as in yeast or mammalian cells needs to be conducted.

Far-Western blotting has been widely applied to detect protein-protein interaction [64]. In the present study, we investigated the interaction between rTs31 and IEC proteins, with results demonstrating that approximately 27 bands of IEC proteins bonded with rTs31. As shown in Fig. 4, the binding of rTs31 with IECs was dose-dependent on both rTs31 and IEC proteins. The cellular localization of this binding was further observed by confocal microscopy, and the results indicated the rTs31 was specifically bonded to IECs and entered into the cytoplasm. Other studies indicated that when IL1 were co-cultured with IECs, the larvae produced several proteases which entered the IECs [21, 65]. Furthermore, anti-rTs31 antibodies partially blocked the IL1 invasion of IECs and the interruption of invasion was dose-dependent on anti-rTs31 antibodies. Our results suggested that Ts31 interacted with IECs, and might promote IL1 invasion of IECs and the establishment of parasitism in the host [66, 67]. However, which IEC proteins bind to Ts31 needs to be further determined by a co-immunoprecipitation assay and mass spectrometry; the mechanism of Ts31-IECs interaction should also be investigated in further experiments.

Vaccination of mice with the rTs31 triggered the specific humoral immune response and significant protection, as demonstrated by the significant reduction in worm burdens of intestinal AW and ML in vaccinated mice after the challenge. The reduction of worm burden observed in the present study is comparable with that of mice vaccinated by recombinant proteins of *T. spiralis* serine proteases TspSP1.2 [23], nudix hydrolase [55], glutathione S-transferase [44] and serine protease inhibitor [68]. The immune protection elicited by vaccination with rTs31 might be due to the production of a high level of anti-Ts31 IgG, which neutralized the ability of serine proteases to hydrolyze the host’s intestinal epithelium and other tissues [24, 69]. Furthermore, our results also demonstrate that anti-rTs31 antibodies noticeably inhibit IL1 invasion of IECs and with inhibition being dose-dependent on anti-Ts31 antibodies. The immune protection induced by rTs31 may also be attributed to the fact that anti-rTs31*Trichinella* IgG can bond to the epicuticle and mouth of this nematode and generate a cap-like immune complex at the worm anterior. This may physically interrupt larval invasion of enterocytes and therefore block IL1 establishment and development in the enteral epithelium [15, 66, 70]. Specific anti-*Trichinella* antibodies perform a crucial action by interfering directly with parasite protein function, facilitate the entrapment of larvae in intestinal mucus and results in worm expulsion from the intestine [71]. Previous studies revealed that anti-*Trichinella* antibodies also participate in the destruction of *T. spiralis* NBL by an ADCC pattern [50, 51]. Therefore, Ts31 might be a potential anti-*Trichinella* vaccine molecular target.

*Trichinella spiralis* is a multicellular parasitic nematode and has a complicated antigenicity and life-cycle. Vaccination of mice with a single recombinant *Trichinella* protein only elicited a partial protection against larval challenge. Thus, multi-epitope vaccines against various *T. spiralis* stage worms should be explored [14, 16, 72]. Since the protein antigenicity in the stomach will be reduced significantly [73], oral vaccination with Ts31 DNA vaccine is likely to be a more suitable vaccination strategy to elicit long-term intestinal mucosal immune responses against the early invasive worms during enteral *Trichinella* infection [74–77], based on the route of *Trichinella* infection (eating raw or undercooked meat). Therefore, oral polyvalent anti-*Trichinella* vaccines should be developed in future study.

## Conclusions

Ts31 was expressed at different life-cycle stages of *Trichinella spiralis* and located principally at the cuticle and stichosome of the parasite. rTs31 was capable of specially bonding to IECs, and the binding site was located in the cytoplasm of the IECs. Immunization of mice with rTs31 generated a significant humoral immune response and protection, as demonstrated by a significant reduction of intestinal AW and ML burden after larval challenge. Anti-rTs31 antibodies inhibited *T. spiralis* invasion of the host’s enterocytes in a dose-dependent pattern, and participated in the killing of the NBL *via* ADCC. Our results indicate that there might be an interaction between Ts31 and IECs. The Ts31 participated in the IL1 invasion of intestinal epithelium and could be a candidate vaccine target molecule against *T. spiralis* infection.

## Abbreviations

ADCC: Antibody-dependent cellular cytotoxicity
AW: Adult worms
DAB: Diaminobenzidine
dpi: Days post-infection
ES: Excretory/secretory
FBS: Fetal bovine serum
IECs: Intestinal epithelial cells
IIF: Indirect immunofluorescence
IL1: Intestinal L1 larvae
LPG: Larvae per gram
MBP: Maltose-binding protein
ML: Muscle larvae
NBL: Newborn larvae
OPD: O-phenylenediamine dihydrochloride
ORF: Open reading frame
PBST: PBS-0.5% Tween 20
PECs: Peritoneal exudates cells
PI: Propidium iodide
qPCR: Quantitative PCR
SD: Standard deviation
Ts31: *Trichinella spiralis* 31 kDa protein
